# Rhizobium Inoculation Enhances the Resistance of Alfalfa and Microbial Characteristics in Copper-Contaminated Soil

**DOI:** 10.3389/fmicb.2021.781831

**Published:** 2022-01-12

**Authors:** Chengjiao Duan, Yuxia Mei, Qiang Wang, Yuhan Wang, Qi Li, Maojun Hong, Sheng Hu, Shiqing Li, Linchuan Fang

**Affiliations:** ^1^State Key Laboratory of Soil Erosion and Dryland Farming on the Loess Plateau, Institute of Soil and Water Conservation, Chinese Academy of Sciences & Ministry of Water Resources, Yangling, China; ^2^University of Chinese Academy of Sciences, Beijing, China; ^3^State Key Laboratory of Agricultural Microbiology, College of Life Science and Technology, Huazhong Agricultural University, Wuhan, China; ^4^College of Natural Resources and Environment, Northwest A&F University, Yangling, China; ^5^College of Urban and Environmental Sciences, Central China Normal University, Wuhan, China; ^6^College of Agronomy, Northwest A&F University, Yangling, China; ^7^Chinese Academy of Sciences (CAS), Center for Excellence in Quaternary Science and Global Change, Xi’an, China

**Keywords:** Cu-stress, alfalfa, plant resistance, enzyme activity, symbiosis system

## Abstract

Some studies have reported the importance of rhizobium in mitigating heavy metal toxicity, however, the regulatory mechanism of the alfalfa-rhizobium symbiosis to resist copper (Cu) stress in the plant-soil system through biochemical reactions is still unclear. This study assessed the effects of rhizobium (*Sinorhizobium meliloti* CCNWSX0020) inoculation on the growth of alfalfa and soil microbial characteristics under Cu-stress. Further, we determined the regulatory mechanism of rhizobium inoculation to alleviate Cu-stress in alfalfa through plant-soil system. The results showed that rhizobium inoculation markedly alleviated Cu-induced growth inhibition in alfalfa by increasing the chlorophyll content, height, and biomass, in addition to nitrogen and phosphorus contents. Furthermore, rhizobium application alleviated Cu-induced phytotoxicity by increasing the antioxidant enzyme activities and soluble protein content in tissues, and inhibiting the lipid peroxidation levels (i.e., malondialdehyde content). In addition, rhizobium inoculation improved soil nutrient cycling, which increased soil enzyme activities (i.e., β-glucosidase activity and alkaline phosphatase) and microbial biomass nitrogen. Both Pearson correlation coefficient analysis and partial least squares path modeling (PLS-PM) identified that the interactions between soil nutrient content, enzyme activity, microbial biomass, plant antioxidant enzymes, and oxidative damage could jointly regulate plant growth. This study provides comprehensive insights into the mechanism of action of the legume-rhizobium symbiotic system to mitigate Cu stress and provide an efficient strategy for phytoremediation of Cu-contaminated soils.

## Introduction

Heavy metal effluence of the soil is a severe issue that directly interrupts environmental resources, ecosystem, and food safety (Sahito et al., 2021; Wang J. et al., 2021). Of these pollutants, copper (Cu) has become one of the main heavy metal contaminants because of its common use in industry and agriculture such as in mining, metal processing, fertilizers, pesticides, and municipal wastes (Huang et al., 2018; Saxena et al., 2020). Cu-contaminated soil displays severe phytotoxic effects, for example, plant growth retardation, nutrient disturbances, and interference with several metabolic processes (Chen et al., 2015). Moreover, Cu-contamination not only causes phytotoxicity but also endangers soil enzyme activities, and damages soil environmental health, which is ultimately detrimental to human health (Chen et al., 2019, 2021).

Cu ions, which are readily absorbed by plant roots, compete with each other and are transferred to other organs (Sun et al., 2015; Duan et al., 2018). Excessive Cu concentration can inhibit plant growth, lead to nutrient deficiencies, reduce antioxidant enzyme activity, and cause oxidation stress by producing of reactive oxygen species (ROS) (Liu et al., 2016; Abbas et al., 2017). Malondialdehyde (MDA) is the final product of lipids peroxidation, and its content in plants can be an excellent indicator of plant damage (Beiyuan et al., 2021). Generally, plants directly or indirectly trigger enzymatic antioxidants [comprising of superoxide dismutase (SOD), peroxidase (POD), catalases (CAT), and ascorbate peroxidase (APX)] to balance elevated ROS and MDA levels, thereby alleviating metal-induced oxidative stress and promoting plant growth. Soil enzymes are extremely sensitive to changes in the soil environment, and they are crucial participants in soil nutrient cycles as well as functional sustainability (Duan et al., 2018; Aponte et al., 2020). Excessive metal concentrations have serious detrimental effects on soil enzyme activity and microorganisms, primarily in terms of soil basal respiration, microbial biomass, enzymatic activity, and soil microbial diversity (Molina-Santiago et al., 2019; Xiao et al., 2020). To date, soil enzyme activities have been universally used as an indicator of the ecological health of terrestrial ecosystems under heavy metal contamination (Wang et al., 2019; Hu et al., 2021). Yang et al. (2016) found that catalase is able to decompose H_2_O_2_ and protect organisms against damage. Moreover, some researchers have reported that phosphatases play an important role in the decomposition of organic phosphorus (P) compounds and have a very important role in improving soil quality (Yang et al., 2019; Wang et al., 2020). Therefore, restoration of soil oxidase and nutrient cycling enzyme activities may be essential for soil health. In this case, plant growth can improve the microbial properties of metal-contaminated soils by root activity their or root secretions. However, the lower nutrient content and higher toxicity of the contaminated soil form two major factors limiting plant growth (Ju et al., 2019; Huang et al., 2020). Therefore, to promote plant growth and reduce potential health risks, there is an urgent need to explore effective strategies that can improve plant resistance and enhance soil quality.

Recently, legumes have attracted interest for their roles in alleviating toxicity to plants in metal-polluted soils (Ju et al., 2020; Basile and Lepek, 2021). The symbiotic relationship between legumes and beneficial rhizobacteria such as plant-growth-promoting-rhizobacteria (PGPRs), can directly or indirectly promote plant growth by increasing nitrogen (N) and P uptake, and by enhancing plant defenses in heavy metal-contaminated soils (He et al., 2020). PGPRs are found in a large group of microorganisms that can interact with plants and promote plant growth, including *Azotobacter*, *Bacillus*, *Pseudomonas*, and *Rhizobium*, etc. (Ferreira et al., 2019). Therefore, as a novel phytobacterial strategy, PGPRs have been used to promote plant growth in heavy metal contaminated soils and enhance soil quality. Ju et al. (2019) showed that inoculation with PGPRs increased the activity of soil enzymes such as urease and β-glucosidase compared to the uninoculated control, which in turn improved soil quality. Moreover, Kong Z. et al. (2015), Kong Z. Y. et al. (2015), and Chen et al. (2018) reported that alfalfa-rhizobium symbiosis can promote plant growth and regulate antioxidant enzyme activity through the synthesis of indoleacetic acid and iron carriers. Allito et al. (2020, 2021) studied the effect of legume-rhizobium symbiosis on the biomass, nodulation, nitrogen fixation and nutrient uptake efficiency of faba bean. However, the soil-plant system is a whole, and the detailed mechanism of legume-rhizobium mediated mitigation of Cu-stress via the biochemical response of the plant-soil system is still limited.

Alfalfa (*Medicago sativa*) is a perennial forage grass that can survive in extreme environments and is widely distributed in the mining areas of northwest China (Wang et al., 2016; Peng et al., 2020). Therefore, it is important to further improve the resistance of alfalfa to heavy metals, reduce their accumulation in plant tissues, and increase the biomass of pastures for safe livestock production. In this study, a Cu-resistant strain (*Sinorhizobium meliloti*) was selected as an exogenous additive to investigate the effects of rhizobium on the growth and physiology of alfalfa in Cu-contaminated soil. We explored the effect of rhizobium inoculation on the microbial properties of Cu-contaminated soil. Further, this study determined the regulatory mechanism of rhizobium inoculation to alleviate Cu-stress in alfalfa through plant-soil system. The results of this study will enhance our understanding of the mechanisms associated with microbial inoculation in promoting plant resistance to Cu stress and providing a new strategy for the phytoremediation of Cu-contaminated soil.

## Materials and Methods

### Experimental Design

Soil samples (obtained from the top 20-cm layer) were collected from a farmland in Yangling District, Shaanxi Province, China. The soil properties were as follows: pH, 8.49; total Cu, 13.2 mg kg^–1^; soil organic carbon (SOC), 7.65 g kg^–1^; total nitrogen (TN), 0.20 g kg^–1^; total phosphorus (TP), 0.36 g kg^–1^; available phosphorus (AP), 27.5 mg kg^–1^; cation exchange capacity (CEC), 16.42 cmol kg^–1^. The collected soil was air-dried, cleared of stones and plant debris, and sieved. Thereafter, CuSO_4_ solution was added to the soil and mixed thoroughly, and maintained in dark at 25°C for 3 months. Ultimately, the experiment included five Cu-addition levels: 0, 200, 400, 600, and 800 mg kg^–1^, respectively (denoted as Cu 0, Cu 200, Cu 400, Cu 600, and Cu 800, respectively).

The prepared Cu-contaminated soil was mixed and passed through a 2 mm sieve, and then the soil (600 g) was packed into plastic pots with a diameter of 10 cm. The soil was balanced for 1 week while maintaining at 60% of the water holding capacity. Alfalfa (*M. sativa*) seeds were sterilized with hydrogen peroxide solution (30%, v:v) and washed three times with distilled water. The seeds were placed in seedling trays containing germination paper and incubated to await seed germination for 72 h, with the germination paper kept moist. Thereafter, 20 seedlings showing the same growth characteristics were selected and transplanted into each pot at a depth of 5 mm. After the alfalfa grew its first leave, the plant roots were injected with a rhizobial bacterial solution; in this study, a metal-resistant rhizobial strain *Sinorhizobium meliloti* CCNWSX0020 was used (Fan et al., 2011). The source and growth of *S. meliloti* has been described in our previous study (Duan et al., 2019). The *S. meliloti* suspensions (20 mL) were added to the plant roots each week (three times in total); in uninoculated controls, same amount of sterile distilled water was used. For the rest of the time, the soil moisture content was maintained at about 60%. Each treatment had three replicates. Finally, a total of 10 treatments: Cu 0, Cu 0 + *S. meliloti*, Cu 200, Cu 200 + *S. meliloti*, Cu 400, Cu 400 + *S. meliloti*, Cu 600, Cu 600 + *S. meliloti*, Cu 800, and Cu 800 + *S. meliloti*. Plants and soils were harvested on day 60 after the first inoculation, and indicators such as plant and soil enzyme activities were measured.

### Determination of Plant Growth Indexes and Copper Contents

The chlorophyll content in plant leaves was extracted with 90% acetone, and then quantified using a spectrophotometer (UV3200, Shimador, Japan) (Richardson et al., 2002; Fang et al., 2020). A straightedge was used to measure alfalfa shoot height and root length. The shoots and roots were washed with sterilized deionized water and dried at 60°C to a constant weight. The shoot and root samples digested with H_2_SO_4_ and H_2_O_2_ were diluted with distilled water, and analyzed for N and P contents in the tissue using a flow analyzer (AA3, SEAL Company, Germany). Furthermore, approximately 0.3 g alfalfa samples digested with a mixture containing 8 mL HNO_3_ and 2 mL HClO_4_ were diluted to a certain volume (i.e., 100 mL) with distilled water, and the solution was analyzed for Cu concentration using an atomic absorption spectrophotometer (Hitachi, FAAS Z-2000, Japan). The Cu uptake was calculated as follows: Total Cu uptake = shoot/root biomass × shoot/root Cu concentration.

### Determination of Malondialdehyde, Soluble Protein Content, and Antioxidant Enzyme Activities

MDA, soluble protein content, and antioxidant enzyme activities in alfalfa were determined using fresh shoots and roots. The MDA content in alfalfa tissues was determined using a kit provided by Suzhou Comin Biotechnology Co., Ltd. (Suzhou, China). The procedure was as follows: alfalfa shoot and root were ground using 1 mL extract. The mixture was centrifuged at 8,000 *g* for 15 min. The supernatant was aspirated and added to the color development solution, and placed in a boiling water bath for 30 min. After cooling and centrifugation, the MDA content in the supernatant was then determined by a microplate reader (Spark, TecanGroup, Ltd.). The soluble protein content was determined according to the manufacturer’s instructions.

Plant antioxidant enzyme activities (i.e., SOD, POD, CAT, and APX) were also determined using the purchased kits. Fresh alfalfa tissue samples were ground with 1 mL extract and centrifuged at 8,000 *g* for 15 min. The supernatant was analyzed spectrophotometrically after treating it with the appropriate chromogenic agents corresponding to each enzyme (Chen et al., 2018).

### Assays of Soil Physicochemical Properties

Soil pH was determined by conventional methods, and the detailed steps were referred to the previous study (Duan et al., 2018). The cation exchange capacity (CEC) was determined by ammonium acetate (NH_4_OAc) method. The SOC and TN were determined using the K_2_CrO_7_–H_2_SO_4_ oxidation and Kjeldahl methods, respectively. Air-dried soils were digested with H_2_SO_4_ and HClO_4_ were analyzed for TP at 880 nm using an ultraviolet spectrophotometer (UV3200, Shimador, Japan). Soil NH_4_^+^-N and NO_3_^–^-N were determined using a continuous-flow autoanalyzer. Dissolved organic carbon (DOC) in fresh soil samples was extracted with 50 mL distilled water and then measured using a LiquiTOCII analyzer (Elementar, Germany). Available P (AP) was determined by sodium bicarbonate extraction method. The total soil Cu content was determined using the modified USEPA Method 3051A, with the following procedure: the soil samples (sieved to 0.149 mm) were digested with 15 mL acid mixture (i.e., 8 mL HNO_3_, 2 mL HClO_4_, and 5 mL HCl) and diluted with distilled water. Finally, the Cu concentration was determined using an atomic absorption spectrophotometer.

### Assays of Soil Enzyme Activities and Microbial Biomass

Soil enzyme activity, including urease, β-glucosidase, alkaline phosphatase, and catalase was measured as described by Guan (1986) and Duan et al. (2018). The urease, β-glucosidase and alkaline phosphatase activities were determined using a spectrophotometer at 587, 400 and 578 nm, respectively. Among them, urease, was used by the sodium phenolate colorimetric method, β-glucosidase by the *p*-nitrophenol colorimetric method, and alkaline phosphatase by the sodium benzyl phosphate colorimetric method. Moreover, soil catalase activity was determined by titrating with potassium permanganate.

Microbial biomass carbon (MBC) and nitrogen (MBN) were analyzed using a previously described method with slight modifications (Vance et al., 1987; Wang et al., 2020). Fresh soil samples (10 g) were fumigated with ethanol-free chloroform in dark for 24 h and extracted with 0.5 M potassium sulfate solution (40 mL) for 30 min. The supernatant was filtered, and the extractable organic carbon (EOC) and nitrogen (EON) contents were determined simultaneously using a LiquiTOC II analyzer (Elementar, Germany). Similarly, EOC and EON were estimated in 10 g of unfumigated soil samples. MBC and MBN were calculated from the difference in EOC and EON between fumigated and non-fumigated samples, respectively.

### Statistical Analyses

A two-way analysis of plant and soil properties between different treatments was performed using IBM SPSS 20.0 software and Duncan’s *post-test* (*P* < 0.05) was used for multiple testing. In addition, the difference between non-inoculated and inoculated alfalfa under the same Cu concentration condition was examined by *T*-test. All bar graphs were plotted using Origin Pro 2021. Pearson correlation coefficient analysis and partial least squares path modeling (PLS-PM) analyses were performed using the software packages “ggcorrplot” and “plspm” in R (version 3.6.2). The relationships between different indicators were compared and determined using the correlation heat map, and PLS-PM was used to determine the direct and indirect factors affecting plant growth.

## Results

### Plant Growth Phenotype and the N and P Content of Alfalfa

**FIGURE 1:**
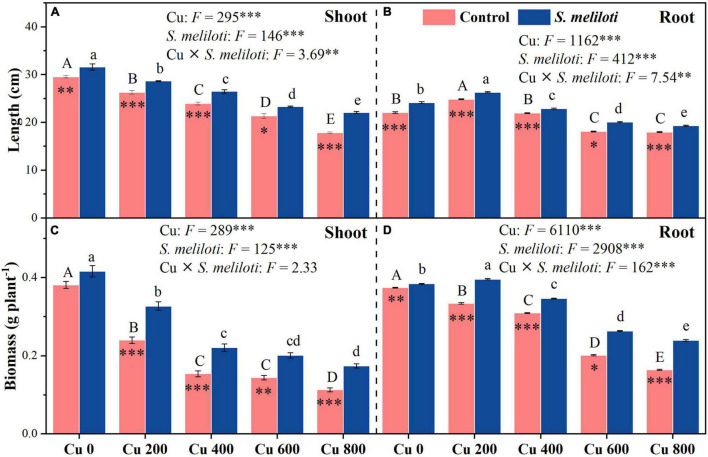
The effect of inoculation with *S. meliloti* on length and biomass in the shoot (left panel) and root (right panel) of alfalfa with different Cu concentration treatments. The capitalized (Cu) and lowercase (*S. meliloti*) letters above the bars indicate significant difference between different Cu concentrations (*P* < 0.05). The asterisks indicate significant differences between non-inoculated and inoculated alfalfa under the same Cu concentration condition. Each value represents the mean ± SE (*n* = 3). ***P < 0.001; **P < 0.01; *P < 0.05.

As shown in Figure 1, the shoot and root length, and biomass of alfalfa decreased with increasing Cu concentration in the control, but rhizobium inoculation alleviated the growth inhibition of alfalfa. In addition, Cu concentration and rhizobium inoculation had significant main and interactive effects on the shoot and root length, and root biomass (*P* < 0.01; Figure 1). The total chlorophyll content increased significantly after rhizobium inoculation, and this increase was prominent at a Cu concentration of 800 mg kg^–1^, which was 1.44 times higher than that of the uninoculated control (Table 1). Cu concentration and rhizobium inoculation had significant interactive effects on total chlorophyll and chlorophyll *a* (*P* < 0.05).

**TABLE 1:** The chlorophyll, nitrogen and phosphorus content in alfalfa tissue.

Treatments		Chlorophyll content (mg g^–1^)						Nitrogen (g kg^–1^)				Phosphorus (g kg^–1^)			
		a		b		Total		Shoot		Root		Shoot		Root	
Cu 0	Control	0.62 ± 0.01 Ab		0.42 ± 0.02 Aa		1.04 ± 0.02 ABb		21.1 ± 0.37 Ab		17.5 ± 0.17 Aa		1.25 ± 0.01 Ab		0.81 ± 0.01 Ab	
	*S. meliloti*	0.66 ± 0.02 BCa		0.47 ± 0.03 Aa		1.13 ± 0.03 Ba		23.1 ± 0.41 Aa		17.2 ± 0.18 Aa		1.51 ± 0.03 Aa		1.33 ± 0.02 Aa	
Cu 200	Control	0.63 ± 0.01 Ab		0.43 ± 0.01 Aa		1.06 ± 0.01 Ab		20.0 ± 0.39 Ba		15.9 ± 0.29 Bb		1.2 ± 0.01 Ba		0.76 ± 0.01 Ba	
	*S. meliloti*	0.74 ± 0.02 Aa		0.48 ± 0.02 Aa		1.22 ± 0.03 Aa		21.4 ± 0.93 Ba		17.2 ± 0.23 Aa		1.24 ± 0.01 Ba		0.74 ± 0.02 Ba	
Cu 400	Control	0.56 ± 0.02 Aa		0.41 ± 0.01 Aa		0.97 ± 0.01 Bb		17.1 ± 0.11 Ca		14.3 ± 0.07 Cb		1.09 ± 0.03 Ca		0.60 ± 0.01 Ca	
	*S. meliloti*	0.62 ± 0.01 Ca		0.42 ± 0.01 ABa		1.04 ± 0.01 Ca		17.8 ± 0.30 Ca		15.2 ± 0.09 Ca		0.97 ± 0.02 Db		0.63 ± 0.01 Ca	
Cu 600	Control	0.61 ± 0.03 Aa		0.40 ± 0.02 Aa		1.01 ± 0.04 ABb		17.7 ± 0.32 Ca		14.4 ± 0.12 Cb		0.90 ± 0.01 Db		0.59 ± 0.01 Cb	
	*S. meliloti*	0.71 ± 0.01 ABa		0.45 ± 0.01 Aa		1.16 ± 0.01 ABa		18.4 ± 0.05 Ca		15.6 ± 0.02 Ca		0.97 ± 0.02 Da		0.64 ± 0.02 Ca	
Cu 800	Control	0.38 ± 0.04 Bb		0.22 ± 0.01 Bb		0.61 ± 0.02 Cb		17.1 ± 0.18 Cb		14.7 ± 0.15 Cb		0.74 ± 0.01 Eb		0.51 ± 0.01 Db	
	*S. meliloti*	0.52 ± 0.03 Da		0.37 ± 0.01 Ba		0.88 ± 0.02 Da		18.9 ± 0.38 Ca		16.2 ± 0.07 Ba		1.05 ± 0.02 Ca		0.55 ± 0.02 Da	
Factor (Df)		*F*	*P*	*F*	*P*	*F*	*P*	*F*	*P*	*F*	*P*	*F*	*P*	*F*	*P*
Cu (4)		28.9	***	37.1	***	94.3	***	47.7	***	94.6	***	282	***	788	***
*S. meliloti* (1)		32.0	***	42.1	***	107	***	25.2	***	82.9	***	106	***	334	***
Cu × *S. meliloti* (4)		4.40	*	1.51	NS	6.50	**	1.15	NS	9.27	***	51.5	***	221	***

The capitalized letters indicate significant differences between different Cu concentrations, whereas the lower-case letters indicate significant differences between non-inoculated and inoculated alfalfa under the same Cu concentration condition (P < 0.05). Each value represents the mean ± SE (n = 3). ^***^P < 0.001; ^**^P < 0.01; *P < 0.05; NS, no significant.

The N level in alfalfa shoots and roots was higher in the treatment without Cu, but did not differ significantly with increasing Cu stress (Table 1). Rhizobium inoculation increased the shoot N content at the Cu concentrations of 0 and 800 mg kg^–1^. The root N content increased significantly with rhizobium inoculation. In addition, Cu concentration and rhizobium inoculation had significant main and interactive effects on the P content in the plant shoots and roots (*P* < 0.001; Table 1). Compared to the uninoculated control, rhizobium inoculation with significantly increased the P levels in the alfalfa tissues, particularly at Cu 600 and Cu 800 treatments (*P* < 0.05).

### Differences in Copper Concentration and Copper Uptake by Alfalfa

Rhizobial effect on Cu concentrations varied in different parts of alfalfa (Table 2). Notably, exogenous addition of rhizobium significantly reduced the Cu concentration in the shoots, but noticeably increased it in the roots (*P* < 0.05). In the shoots, the Cu uptake by the inoculated plants was evidently lower than that of the uninoculated plants in the Cu 0, Cu 400, and Cu 600 treatments (*P* < 0.05). Furthermore, rhizobium inoculation decreased the Cu uptake in roots when treated with low Cu concentrations (0 or 200 mg kg^–1^). However, under high Cu concentrations, Cu uptake in roots increased with rhizobium inoculation. Except for the Cu 0 treatment, the Cu transfer coefficients were considerably less than 1.0. Rhizobium inoculation increased the Cu transfer coefficients in treatments with low Cu concentrations (0 or 200 mg kg^–1^). However, under high Cu concentrations, the Cu transfer coefficients decreased with rhizobium inoculation.

**TABLE 2:** Cu concentrations and the total uptake of Cu in alfalfa tissue.

Treatments		Cu concentrations (mg kg^–1^)				Total uptake (μg plant^–1^)				TF	
		Shoot		Root		Shoot		Root			
Cu 0	Control	8.54 ± 0.07 Da		24.9 ± 0.56 Ea		3.25 ± 0.03 Ba		9.30 ± 0.21 Da		0.34 ± 0.01 Ab	
	*S. meliloti*	6.45 ± 0.12 Db		3.06 ± 0.22 Eb		2.68 ± 0.05 Bb		1.18 ± 0.08 Eb		2.13 ± 0.19 Aa	
Cu 200	Control	12.3 ± 0.90 Ca		42.8 ± 0.92 Da		2.96 ± 0.22 Ba		14.3 ± 0.31 Ca		0.29 ± 0.03 Bb	
	*S. meliloti*	10.4 ± 0.15 Ba		26.1 ± 0.04 Db		3.41 ± 0.05 Aa		10.3 ± 0.02 Db		0.40 ± 0.01 Ba	
Cu 400	Control	15.9 ± 0.27 Ba		50.4 ± 0.33 Cb		2.45 ± 0.04 Ca		15.6 ± 0.10 Bb		0.32 ± 0.01 Ba	
	*S. meliloti*	8.54 ± 0.08 Cb		64.6 ± 0.32 Ca		1.89 ± 0.02 Cb		22.4 ± 0.11 Ba		0.13 ± 0.01 Ca	
Cu 600	Control	28.7 ± 0.56 Aa		93.3 ± 1.20 Ab		4.13 ± 0.08 Aa		18.8 ± 0.24 Ab		0.31 ± 0.01 Ba	
	*S. meliloti*	15.7 ± 0.70 Ab		119 ± 0.75 Aa		3.16 ± 0.14 Ab		31.5 ± 0.20 Aa		0.13 ± 0.01 Cb	
Cu 800	Control	13.2 ± 0.70 Ca		55.2 ± 2.89 Bb		1.49 ± 0.08 Da		9.06 ± 0.47 Db		0.24 ± 0.02 Ca	
	*S. meliloti*	7.50 ± 0.70 CDb		83.1 ± 0.68 Ba		1.31 ± 0.12 Da		19.9 ± 0.16 Ca		0.09 ± 0.01 Cb	
Factor (Df)		*F*	*P*	*F*	*P*	*F*	*P*	*F*	*P*	*F*	*P*
Cu (4)		230	***	2,025	***	157	***	2,145	***	108	***
*S. meliloti* (1)		332	***	72.3	***	33.2	***	642	***	51.5	***
Cu × *S. meliloti* (4)		38.2	***	229	***	14.2	***	825	***	96.6	***

The capitalized letters indicate significant differences between different Cu concentrations, whereas the lower-case letters indicate significant differences between non-inoculated and inoculated alfalfa under the same Cu concentration condition (P < 0.05). TF (translocation factor: shoot concentration/root concentration). Each value represents the mean ± SE (n = 3). ^***^P < 0.001.

### Plant Oxidative Damage, Soluble Protein, and Antioxidant Enzyme Activity

**FIGURE 2:**
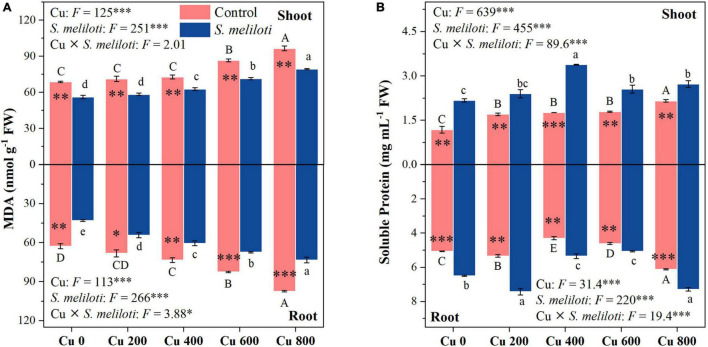
The effect of inoculation with *S. meliloti* on MDA **(A)** and soluble protein **(B)** in shoot (top panel) and root (lower panel) of alfalfa with different Cu concentration treatments. MDA: malondialdehyde. The capitalized (Cu) and lowercase (*S. meliloti*) letters above the bars indicate significant difference between different Cu concentrations (*P* < 0.05). The asterisks indicate significant differences between non-inoculated and inoculated alfalfa under the same Cu concentration condition. Each value represents the mean ± SE (*n* = 3). ***P < 0.001; **P < 0.01; *P < 0.05.

**FIGURE 3:**
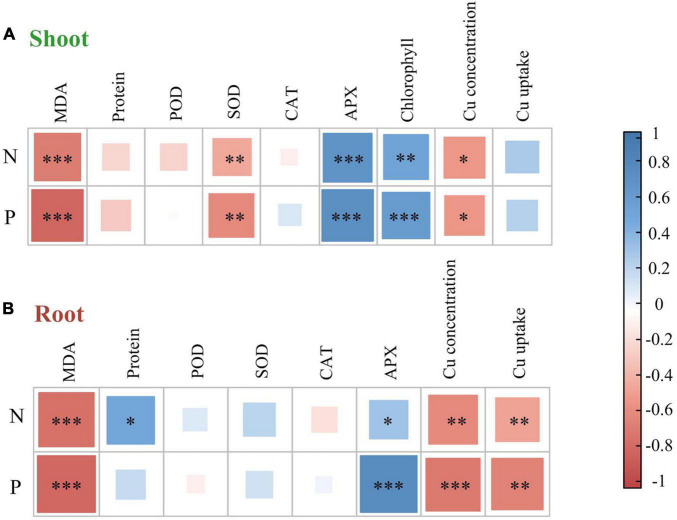
A correlation heat map illustrating pairwise relationships between plant nutrient elements and plant properties in alfalfa shoot **(A)** and root **(B)** based on Pearson correlation coefficient analysis. N, nitrogen concentration; P, phosphorus concentration; MDA, malondialdehyde; Protein, soluble protein; SOD, superoxide dismutase; POD, guaiacol peroxidase; CAT, catalase; APX, ascorbate peroxidase. ***P < 0.001; **P < 0.01; *P < 0.05.

The effect of heavy metals on lipid peroxidation damage was evaluated by measuring the MDA content (Figure 2A), which increased in both shoots and roots as the Cu concentration increased during treatments, but rhizobium inoculation reduced MDA accumulation in plant tissues. In the same Cu concentration treatment, the reduction of shoot MDA content after inoculation with rhizobium was less than that of the root. Moreover, Cu concentration and rhizobium inoculation had significant interactive effects on the root MDA content (*P* < 0.05). This indicates that rhizobium inoculation had a relatively higher relieving effect on roots than on the aboveground parts. Additionally, MDA content in shoots and roots was negatively correlated with N and P content (*P* < 0.001; Figure 3). Cu concentration and rhizobium inoculation had significant main and interactive effects on the soluble protein content in the plant shoots and roots (*P* < 0.001, Figure 2B). In addition, soluble protein content in roots was significantly positively correlated with N content (*P* < 0.05; Figure 3B).

**FIGURE 4:**
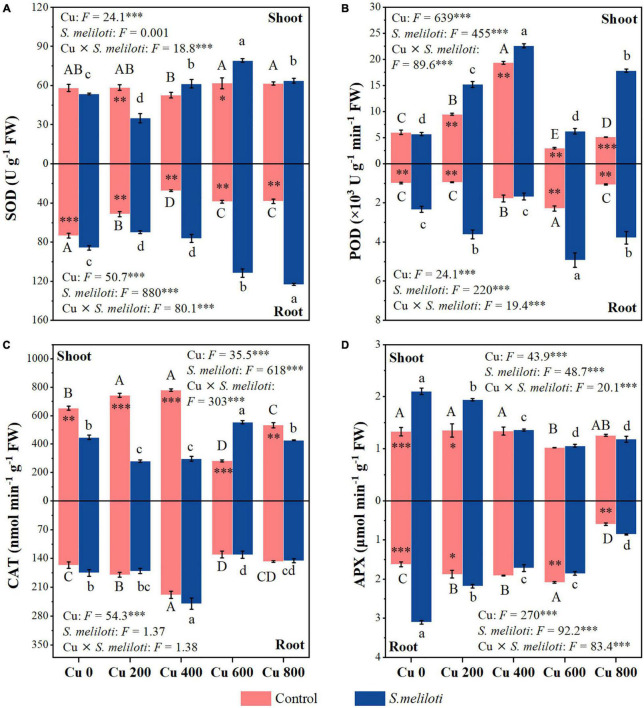
The effect of inoculation with *S. meliloti* on plant antioxidant enzyme activities in alfalfa tissue. SOD, superoxide dismutase; POD, guaiacol peroxidase; CAT, catalase; APX, ascorbate peroxidase. The capitalized (Cu) and lowercase (*S. meliloti*) letters above the bars indicate significant difference between different Cu concentrations (*P* < 0.05). The asterisks indicate significant differences between non-inoculated and inoculated alfalfa under the same Cu concentration condition. Each value represents the mean ± SE (*n* = 3). ***P < 0.001; **P < 0.01; *P < 0.05.

The SOD activity in the shoots of uninoculated plants showed no obvious change, while the roots of alfalfa showed a downward trend under Cu stress (Figure 4A). Compared with the uninoculated alfalfa, plants inoculated with rhizobium exhibited higher SOD activity in the roots (*P* < 0.01; Figure 4A); for example, in the Cu 600 and Cu 800 treatments, root SOD was 2.87 and 3.23 times higher after rhizobia inoculation than in the uninoculated treatment, respectively (Figure 4A). Under the Cu 600 treatment, there was the highest shoot POD activity but lower in the root, suggesting different response mechanisms in the shoot and root (Figure 4B). Cu concentration and rhizobium inoculation had significant main and interactive effects on the POD activity in the plant shoots and roots (*P* < 0.001). Except for Cu 600 treatment, rhizobium inoculation significantly reduced CAT activity in the shoot (*P* < 0.01; Figure 4C). Rhizobium inoculation had no obvious effect on CAT activity in alfalfa roots, and no significant interactive effects between Cu and rhizobium inoculation on root CAT. Differences in APX activity in alfalfa tissue between uninoculated and inoculated plants were inconsistent and influenced by specific Cu treatments (Figure 4D). In addition, the application of rhizobium increased the alfalfa APX activity at low Cu concentrations (0 or 200 mg kg^–1^). SOD activity in shoots was significantly and negatively correlated with N and P content (*P* < 0.01). However, the APX activity of alfalfa (shoot and root) was significantly and positively correlated with the content of N and P, respectively (*P* < 0.05; Figure 3).

### Soil Nutrient, Enzyme Activities, and Microbial Biomass

Soil Cu concentration decreased significantly after rhizobium inoculation (*P* < 0.05; Supplementary Table 1). Cu concentration and rhizobium inoculation had significant main and interactive effects on the NO_3_^–^-N and NH_4_^+^-N contents (*P* < 0.001). The highest AP content was observed in the Cu 600 treatment in both the inoculated and uninoculated treatments. However, rhizobium inoculation did not evidently affect AP. Rhizobium inoculation increased the DOC content at low Cu concentrations (200 or 400 mg kg^–1^), whereas the DOC was significantly reduced at a Cu concentration of 600 mg kg^–1^ (*P* < 0.05). Soil total N and P contents were only influenced by soil Cu concentration and did not respond significantly to rhizobium inoculation. Cu concentration and rhizobium inoculation had significant main and interactive effects on pH and CEC (*P* < 0.01). Moreover, the pH value decreases with increasing Cu concentration.

**FIGURE 5:**
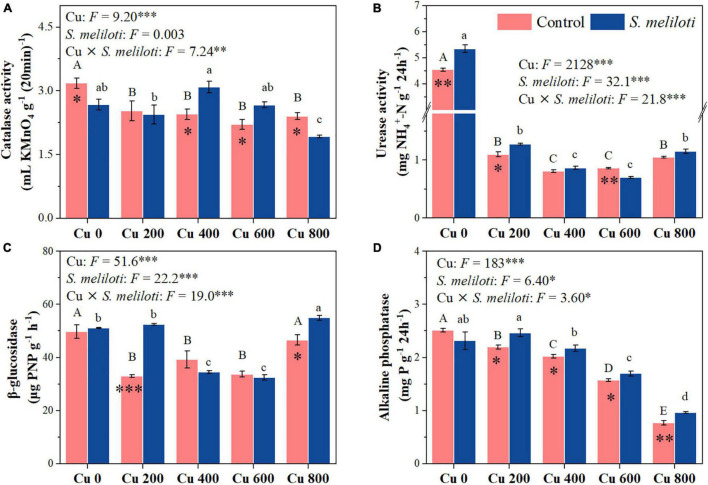
The effect of inoculation with *S. meliloti* on soil enzyme activities under different Cu concentration treatments. The capitalized (Cu) and lowercase (*S. meliloti*) letters above the bars indicate significant difference between different Cu concentrations (*P* < 0.05). The asterisks indicate significant differences between non-inoculated and inoculated alfalfa under the same Cu concentration condition. Each value represents the mean ± SE (*n* = 3). ***P < 0.001; **P < 0.01; *P < 0.05.

**FIGURE 6:**
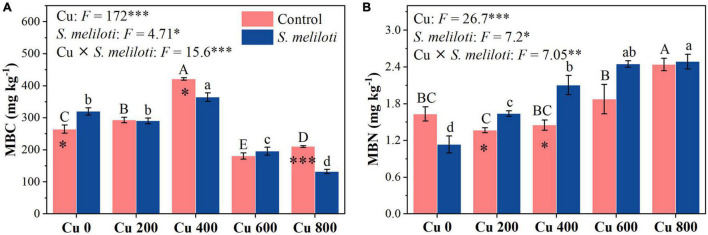
The effect of inoculation with *S. meliloti* on soil microbial biomass carbon and nitrogen under different Cu concentration treatments. MBC, microbial biomass carbon; MBN, microbial biomass nitrogen. The capitalized (Cu) and lowercase (*S. meliloti*) letters above the bars indicate significant difference between different Cu concentrations (*P* < 0.05). The asterisks indicate significant differences between non-inoculated and inoculated alfalfa under the same Cu concentration condition. Each value represents the mean ± SE (*n* = 3). ***P < 0.001; **P < 0.01; *P < 0.05.

The changes in soil enzymes (i.e., catalase, urease, β-glucosidase, and alkaline phosphatase) were shown in Figure 5. In the uninoculated control, the catalase activity in Cu 0 was significantly higher than that in the other treatments, and the increase in Cu concentration strongly inhibited catalase activity, whereas rhizobium inoculation remarkably increased catalase activity in the Cu 400 and Cu 600 treatments (*P* < 0.05). Cu concentration and rhizobium inoculation had significant main and interactive effects on β-glucosidase activity (*P* < 0.001). Furthermore, soil phosphatase activity consistently decreased with increasing Cu concentration, rhizobium inoculation notably affected the alkaline phosphatase activity. However, rhizobium inoculation enhanced urease activity only at low Cu concentrations (0 or 200 mg kg^–1^). Cu concentration and rhizobium inoculation had significant main and interactive effects on MBC and MBN (*P* < 0.05), and rhizobium inoculation significantly increased the MBN content in Cu 200 and Cu 400 treatments (*P* < 0.05; Figure 6).

### Driving Factors Affecting Plant Growth

**FIGURE 7:**
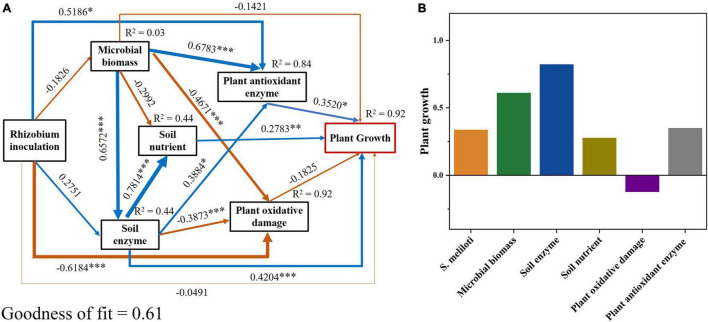
Partial least squares path modeling (PLS-PM) disentangling major pathways of the effects of rhizobium inoculation, soil microbial biomass, enzyme activity, soil nutrient, plant oxidative damage and plant antioxidant enzyme activities on plant growth. Blue and yellow arrows indicate positive and negative flows of causality, respectively. Numbers on the arrow indicate significant standardized path coefficients (***P < 0.001; **P < 0.01; *P < 0.05). *R*^2^ indicates the variance of dependent variable explained by the model. Microbial biomass includes microbial biomass carbon and nitrogen; soil enzyme includes urease, β-glucosidase and phosphatase; soil nutrient includes soil dissolved organic carbon, NO_3_^–^-N and soil available phosphorus; plant oxidative damage includes plant MDA (malondialdehyde); plant antioxidant enzyme includes POD (guaiacol peroxidase), CAT (catalase) and APX (ascorbate peroxidase); plant growth includes the nitrogen and phosphorus contents of tissue.

The N content in shoots and roots showed the highest positive correlations with SOC, DOC, CEC, pH, and soil enzyme activities (*P* < 0.001), and the highest negative correlations with MBN, NH_4_^+^-N, TN, AP, and Cu concentrations (*P* < 0.05; Supplementary Figure 1). In addition, the plant P showed the strongest positive correlation with MBC, TP, SOC, DOC, CEC, pH, and soil enzyme activities (*P* < 0.01), and the strongest negative correlation with MBN, NH_4_^+^-N, TN, AP, and Cu concentrations (*P* < 0.05; Supplementary Figure 1). We used PLS-PM to further reveal the effects of plant physiological indicators, soil physicochemical properties, and soil biochemical indicators on alfalfa growth (Figure 7). The PLS-PM method showed that plant antioxidant enzyme activities, soil nutrient, and soil enzyme activities had a significant positive direct effect (0.35, 0.28, and 0.42, respectively; *P* < 0.01) on plant growth (Figure 7A). The application of *S. meliloti* (0.34), soil microbial biomass (0.61), soil enzyme activities (0.82), soil nutrient (0.28), and antioxidant enzyme activities (0.35) had positive total effects on plant growth, whereas the plant oxidative damage (−0.13) induced negative total effects (Figure 7B).

## Discussion

Plants grown in Cu-contaminated soils may have to endure physiological dysfunction, growth inhibition, reduced biomass, and metal accumulation (Rostami and Azhdarpoor, 2019; Fang et al., 2020). In this study, inoculation of rhizobium (i.e., *S. meliloti*) not only increased the height and biomass of alfalfa, but also increased its chlorophyll content (Table 1 and Figure 1). This indicates that inoculation with rhizobium promoted the photosynthetic reaction rate of plants and alleviated the toxic effects of Cu stress (Chou et al., 2019; Gong et al., 2019). This further confirmed the important role of alfalfa-rhizobium symbiosis in soil Cu bioremediation. Moreover, inoculation with rhizobium significantly reduced the metal transfer coefficient under high Cu-stress (i.e., Cu 600 and Cu 800) (Table 2). This suggests that Cu accumulates mainly in the roots, while a very small amount is transferred to the shoots, which not only facilitates the phytostabilization of Cu, but also improves aboveground forage quality. Rhizobium inoculation significantly increased the N content in alfalfa roots (Table 1), providing a sufficient source of N for plant growth and alleviating alfalfa toxicity. However, the effect of rhizobium inoculation on the N content of the shoots was substantially lower than that of the roots (Table 1). The nodules functioning in the root system are probably the main nitrogen-fixing organs (Stambulska and Bayliak, 2019). This also indicates that the Cu-resistant strain (i.e., *S. meliloti*) can survive at 0–800 mg kg^–1^ Cu concentrations and promote alfalfa nitrogen fixation. Heavy metal tolerant rhizobia affect the biological effectiveness of metals in soil by regulating biological nitrogen fixation and promoting plant growth (Duan et al., 2019; Jian et al., 2019; Shen et al., 2020). Furthermore, P plays an important role in plant growth. Our study found that inoculation with rhizobium had a significant main effect on the P content of alfalfa in the shoot and root parts (Table 1). This may be due to the fact that the addition of rhizobium induced alfalfa roots to secrete more phosphatase, which increased the effectiveness of soil P for alfalfa uptake (Peng et al., 2020). Rhizobia, as a P-dissolving microorganism, lower soil pH by dissolving various small amounts of insoluble P sources, thus increasing P uptake and plant biomass (Ma et al., 2016; Zhou et al., 2020). These results demonstrate that rhizobium inoculation alleviates metal stress by increasing plant nutrient content (N and P) and reducing Cu transfer to the shoot in alfalfa.

Inoculation rhizobium can increase alfalfa growth and improve its resistance to Cu stress. A significant increase in the Cu content of roots after inoculation was observed (Table 2). However, the increased Cu content in roots did not lead to increased content in shoots nor did it inhibiting alfalfa growth, which further indicated that inoculation could improve alfalfa resistance to Cu stress. Although excessive Cu accumulation produced large amounts of MDA in alfalfa seedlings, exacerbating oxidative stress in the cells (Figure 2), inoculation with rhizobium significantly reduced the MDA content in the shoots and roots of the alfalfa. The results of PLS-PM further supported that rhizobium inoculation mitigates oxidative damage caused by Cu toxicity in alfalfa (Figure 7). Oxidative damage and antioxidant enzyme activities can effectively reflect the intensity of toxicity endured by plant cells and are important in understanding the metal-specific toxicity mechanisms in plants (Chen et al., 2018; Romero-Puertas et al., 2018). Kong Z. Y. et al. (2015) suggested that the promotion of plant antioxidant defenses by bacteria may improve symbiotic performance, especially under non-optimal conditions. Previous studies have shown that exogenous nitrogen can alleviate oxidative stress induced by heavy metal exposure (Ju et al., 2019; Wang X. et al., 2021), which further demonstrates that rhizobium-legumes can mitigate oxidative stress through symbiotic nitrogen fixation. In summary, this indicates that rhizobium inoculated plants suffered less oxidative damage and exhibited a higher antioxidant capacity under Cu-stress.

The accumulation of soluble proteins in plants is a response to stress caused by toxic elements, either through the reduction of oxidative stress or the regulation of cellular osmotic capacity (Raza et al., 2020). Rhizobium-inoculated plants exhibited higher soluble protein content in alfalfa tissues compared with uninoculated plants (Figure 2B). This finding indicates that adding rhizobium improved the symbiotic performance of alfalfa and promoted an increase in soluble protein content, especially under non-optimal conditions. Meanwhile, alfalfa has high antioxidant enzyme activities that protect cells from oxidative damage caused by external environmental stresses (i.e., higher MDA contents) (Morina et al., 2016; Hojati et al., 2017). Cu-stress reduced SOD activity in alfalfa tissues (Figure 4A). Cu-contamination-induced destruction of membranes and enzyme systems resulted in reduced SOD activity, especially at high Cu concentrations (600 and 800 mg kg^–1^). Furthermore, rhizobium inoculation considerably affected the increase in SOD activity in alfalfa roots (Figure 4A). This result indicates that rhizobium application enhanced SOD activity in tissues and improved their resistance to stress. Studies have shown that metal resistant beneficial microbes (PGPRs) are often used as bioinoculants to affect metabolic functions and antioxidant enzyme activities of root cells and thus to enhance the establishment, growth and development of remediating plants in metal contaminated soils (Ma et al., 2016; Ju et al., 2020). In comparison with uninoculated alfalfa tissue, the activities of POD and APX in the shoots and roots of inoculated alfalfa increased at different levels of symbiosis (Figure 4). The results showed a significant positive correlation between APX activity, and the N and P contents of alfalfa (Figure 3). In addition, the alfalfa-rhizobium symbiotic system can promote plant nitrogen fixation, stimulate the release of phytohormones and antioxidant enzyme activities, and ultimately reduce the toxicity of alfalfa and promote its growth (Hao et al., 2014; Fang et al., 2020). Nonetheless, the addition of rhizobia reduced the Cu-induced CAT activity in the shoots, except for the Cu 600 treatment (Figure 4C). This suggests that rhizobium inoculation in the shoots inhibits the damage caused by excess hydrogen peroxide; therefore, more CAT activities are unnecessary in eliminating the hydrogen peroxide produced under Cu− concentration in the alfalfa tissues (Cao et al., 2017; Beiyuan et al., 2021). Overall, the above results indicate that rhizobium inoculation responds to Cu-stress with increasing soluble proteins, POD, and APX activities, which therefore alleviates heavy metals toxicity in alfalfa. The way in which rhizobium inoculation assists in increasing plant resistance is the key to revealing the mechanisms that promote growth in inoculation.

Soil enzymes can be used to evolute the effects of the soil remediation process since they relate to the biogeochemical cycling of elements (Yang et al., 2019). Rhizobium inoculation significantly increased catalase activity in the Cu 400 and Cu 600 treatments (Figure 5A). Catalase can participate in the H_2_O_2_ detoxification process by changing the valence reference of heavy metal ions (Duan et al., 2018), and the addition of beneficial bacteria to the soil can further alleviate soil Cu toxicity and increase catalase activity (Yang et al., 2016; Aponte et al., 2020). In addition, soil carbon (C), N and P cycling activities are also associated with microbial metabolism and reflect the interaction between microorganisms and their associative environment (Cui et al., 2019; Ju et al., 2020). Soil β-glucosidase activity is associated with soluble nutrients, such as DOC, and reflects soil C cycling and fertility (Raiesi and Salek-Gilani, 2018). Rhizobium inoculation increased DOC in this study (Supplementary Table 1), and there was a significant positive correlation between DOC and β-glucosidase (Supplementary Figure 1), indicating that rhizobium inoculation promotes soil C cycling and provides nutrients for plant growth. Meanwhile, inoculation with rhizobium significantly promoted alkaline phosphatase activity (Figure 5D). Phosphatase in soils is mainly produced by microbial activity and is critical for mobilizing the organic forms of P (Wei et al., 2019; Ma et al., 2021). These results demonstrated that rhizobium inoculation can promote soil nutrient cycling by stimulating enzyme activities in metal contaminated soil while subsequently providing higher C, N, and P requirements for microbial activity and plant growth. Moreover, the N and P contents of alfalfa were significantly positively correlated with the soil enzymes activities (Supplementary Figure 1), and the PLS-PM results also showed an overall significant positive effect of soil enzymes and microbial biomass on plant growth following *S. meliloti* application (Figure 7). The legume-rhizobium symbiosis regulated the rate of enzyme synthesis via Cu or by altering the interference of toxicity in enzyme-producing microbial community, thus reducing metal toxicity in soil (Yu et al., 2019; Fang et al., 2020). Our results show that heavy metal-resistant soil rhizobacteria can significantly reduce the soil Cu concentrations (Supplementary Table 1), which was favorable for the survival of microorganisms. Based on the above information, we found that the legume-rhizobium symbiotic system promoted alfalfa growth in Cu-contaminated soil by regulating plant physiological and biochemical properties, soil nutrients, and soil enzyme activities.

## Conclusion

This study offers comprehensive insight into how symbiotic microbes affect plant physiology and soil microbial properties to assist alfalfa against Cu stress. Excessive accumulation of Cu in alfalfa exhibited varied toxicity symptoms, including decreased biomass, chlorotic leaves, and inhibition of antioxidant enzyme activities. Rhizobium inoculation significantly improved the growth of alfalfa under Cu-stress by effectively improving the detoxification ability. The legume-rhizobium symbiotic system resists Cu-induced oxidative stress in alfalfa tissues by enhancing antioxidant enzyme activities to scavenge superfluous malondialdehyde. Moreover, rhizobium application improved soil microbial parameters, such as soil enzyme activity and microbial biomass, thereby promoting plant growth. These findings are important for further improve plant resistance and ensure forage and crop production.

## Data Availability Statement

The original contributions presented in the study are included in the article/Supplementary Material, further inquiries can be directed to the corresponding author/s.

## Author Contributions

CD: writing—original draft, writing—review and editing, and visualization. YM, QW, and YW: methodology and writing—review and editing. QL: investigation and methodology. MH and SH: investigation. SL: writing—review and editing. LF: funding acquisition, methodology, resources, supervision, and writing—review and editing. All authors contributed to the article and approved the submitted version.

## Conflict of Interest

The authors declare that the research was conducted in the absence of any commercial or financial relationships that could be construed as a potential conflict of interest.

## Publisher’s Note

All claims expressed in this article are solely those of the authors and do not necessarily represent those of their affiliated organizations, or those of the publisher, the editors and the reviewers. Any product that may be evaluated in this article, or claim that may be made by its manufacturer, is not guaranteed or endorsed by the publisher.
