# Supplementary material for: Rhizobium Inoculation Enhances the Resistance of Alfalfa and Microbial Characteristics in Copper-Contaminated Soil
Source: Front Microbiol. 2022 Jan 12;12:781831. doi: 10.3389/fmicb.2021.781831 (PMC8791600; doi:10.3389/fmicb.2021.781831)
Supplement: Supplementary file 1 [file Table_1.DOCX]

**Rhizobium inoculation enhances the resistance of alfalfa and microbial characteristics in Cu-contaminated soil**

Chengjiao Duan ^a, b^, Yuxia Mei ^e^, Qiang Wang ^d^, Yuhan Wang ^d^, Qi Li ^f^, Maojun Hong ^g^, Sheng Hu ^g^, Shiqing Li ^a^, Linchuan Fang ^a, c*^

^a^ State Key Laboratory of Soil Erosion and Dryland Farming on the Loess Plateau, Institute of Soil and Water Conservation CAS and MWR, Yangling, 712100, China

^b^ University of Chinese Academy of Sciences, Beijing, 100049, China

^c^ CAS Center for Excellence in Quaternary Science and Global Change, Xi'an, 710061, China

^d^ College of Natural Resources and Environment, Northwest A&F University, Yangling, 712100, China

^e^ State Key Laboratory of Agricultural Microbiology, College of Life Science and Technology, Huazhong Agricultural University, Wuhan, 430070, PR China

^f^ College of Urban and Environmental Sciences, Central China Normal University, Wuhan, 430070, PR China

^g^ College of Agronomy, Northwest A&F University, Yangling, 712100, China

* **Corresponding author**

Linchuan Fang (Tel: +86 15249204460, Email: flinc629@hotmail.com)

**Table S1** Effect of rhizobium inoculation on soil physicochemical properties.

| Treatments | | Cu concentration (mg kg^-1^) | | NO_3_^-^-N (mg kg^-1^) | | NH_4_^+^-N (mg kg^-1^) | | TN (g kg^-1^) | | AP (mg mg^-1^) | |
| --- | --- | --- | --- | --- | --- | --- | --- | --- | --- | --- | --- |
| Cu 0 | Control | 8.23 ± 0.36 Ea | | 2.67 ± 0.04 ABb | | 0.44 ± 0.01 Da | | 0.77 ± 0.01 Aa | | 18.3 ± 0.59 Eb | |
|  | *S.meliloti* | 5.34 ± 0.17 Eb | | 2.94 ± 0.07 Ba | | 0.43 ± 0.01 Ca | | 0.76 ± 0.01 Ca | | 22.8 ± 0.67 Da | |
| Cu 200 | Control | 194 ± 0.75 Da | | 2.83 ± 0.03 Aa | | 0.41 ± 0.01 Da | | 0.77 ± 0.01 Aa | | 21.7 ± 0.09 Da | |
|  | *S.meliloti* | 185 ± 0.48 Db | | 2.73 ± 0.03 Ca | | 0.42 ± 0.01 Ca | | 0.78 ± 0.01 ABa | | 21.8 ± 0.19 Da | |
| Cu 400 | Control | 336 ± 1.09 Ca | | 2.71 ± 0.05 ABb | | 1.84 ± 0.01 Ab | | 0.77 ± 0.01 Aa | | 35.8 ± 0.86 Ba | |
|  | *S.meliloti* | 310 ± 3.74 Cb | | 3.22 ± 0.05 Aa | | 2.31 ± 0.01 Aa | | 0.79 ± 0.01 Aa | | 35.5 ± 1.42 Ba | |
| Cu 600 | Control | 536 ± 7.06 Ba | | 2.61 ± 0.08 Ba | | 1.57 ± 0.01 Ba | | 0.78 ± 0.01 Aa | | 38.7 ± 1.08 Aa | |
|  | *S.meliloti* | 505 ± 7.29 Bb | | 2.71 ± 0.04 Ca | | 1.63 ± 0.01 Ba | | 0.78 ± 0.01 ABa | | 39.4 ± 0.34 Aa | |
| Cu 800 | Control | 757 ± 8.76 Aa | | 2.65 ± 0.02 Bb | | 1.21 ± 0.01 Cb | | 0.77 ± 0.01 Aa | | 27.3 ± 1.23 Ca | |
|  | *S.meliloti* | 708 ± 7.16 Ab | | 3.12 ± 0.07 Aa | | 1.63 ± 0.01 Ba | | 0.77 ± 0.01 BCa | | 28.4 ± 0.35 Ca | |
| Factor (Df) | | *F* | *P* | *F* | *P* | *F* | *P* | *F* | *P* | *F* | *P* |
| Cu (4) | | 6479 | *** | 9.70 | *** | 855 | *** | 3.50 | * | 207 | *** |
| *S. meliloti* (1) | | 57.1 | *** | 58.7 | *** | 69.9 | *** | 0.62 | NS | 5.89 | * |
| Cu 🞨 *S. meliloti* (4) | | 6.85 | ** | 12.2 | *** | 21.4 | *** | 1.98 | NS | 2.69 | NS |
|  | | TP  (g kg^-1^) | | SOC (g kg^-1^) | | DOC  (mg kg^-1^) | | pH | | CEC  (cmol kg^-1^) | |
| Cu 0 | Control | 0.81 ± 0.03 ABa | | 6.66 ± 0.11 Aa | | 133 ± 3.93 ABa | | 8.43 ± 0.02 Aa | | 21.1 ± 0.95 ABa | |
|  | *S.meliloti* | 0.84 ± 0.01 Aa | | 6.65 ± 0.20 Aa | | 138 ± 2.80 ABa | | 8.42 ± 0.01 Aa | | 22.5 ± 1.59 ABa | |
| Cu 200 | Control | 0.81 ± 0.01 ABb | | 6.67 ± 0.14 Aa | | 136 ± 0.71 Ab | | 8.40 ± 0.01 Aa | | 18.7 ± 1.13 BCb | |
|  | *S.meliloti* | 0.82 ± 0.01 ABa | | 6.63 ± 0.18 Aa | | 144 ± 2.50 Aa | | 8.25 ± 0.02 Bb | | 24.0 ± 0.95 Aa | |
| Cu 400 | Control | 0.85 ± 0.01 Aa | | 6.54 ± 0.13 Aa | | 97.8 ± 2.24 Cb | | 8.17 ± 0.02 Ba | | 21.6 ± 0.79 Aa | |
|  | *S.meliloti* | 0.82 ± 0.01 ABb | | 6.57 ± 0.09 Aa | | 113 ± 1.47 Da | | 8.16 ± 0.03 Ca | | 19.0 ± 0.74 BCa | |
| Cu 600 | Control | 0.81 ± 0.01 ABb | | 6.75 ± 0.02 Aa | | 135 ± 0.86 Aa | | 8.08 ± 0.02 Ca | | 20.1 ± 0.30 ABCb | |
|  | *S.meliloti* | 0.84 ± 0.01 Aa | | 6.45 ± 0.05 Ab | | 124 ± 1.92 Cb | | 8.00 ± 0.02 Db | | 25.15 ± 0.85 Aa | |
| Cu 800 | Control | 0.78 ± 0.03 Ba | | 6.40 ± 0.07 Aa | | 124 ± 4.52 Ba | | 8.05 ± 0.01 Ca | | 18.3 ± 0.27 Ca | |
|  | *S.meliloti* | 0.79 ± 0.01 Ba | | 6.62 ± 0.04 Aa | | 133 ± 5.74 BCa | | 7.94 ± 0.01 Db | | 18.7 ± 1.30 Ca | |
| Factor (Df) | | *F* | *P* | *F* | *P* | *F* | *P* | *F* | *P* | *F* | *P* |
| Cu (4) | | 3.02 | * | 0.52 | NS | 38.1 | *** | 198 | *** | 5.53 | ** |
| *S. meliloti* (1) | | 0.69 | NS | 0.09 | NS | 6.66 | * | 37.7 | *** | 9.63 | ** |
| Cu 🞨 *S. meliloti* (4) | | 1.29 | NS | 1.22 | NS | 5.27 | ** | 6.01 | ** | 5.72 | ** |

**Note:** TN: total nitrogen; AP: available phosphorus; SOC: soil organic carbon; DOC: dissolved organic carbon; TP: total phosphorus; CEC: cation exchange capacity. The capitalized letters indicate significant differences between different Cu concentrations, whereas the lower-case letters indicate significant differences between non-inoculated and inoculated alfalfa under the same Cu concentration condition (*P* < 0.05). Each value represents the mean ± SE (n = 3). ***, *P* < 0.001; **, *P* < 0.01; *, *P* < 0.05; NS, no significant.


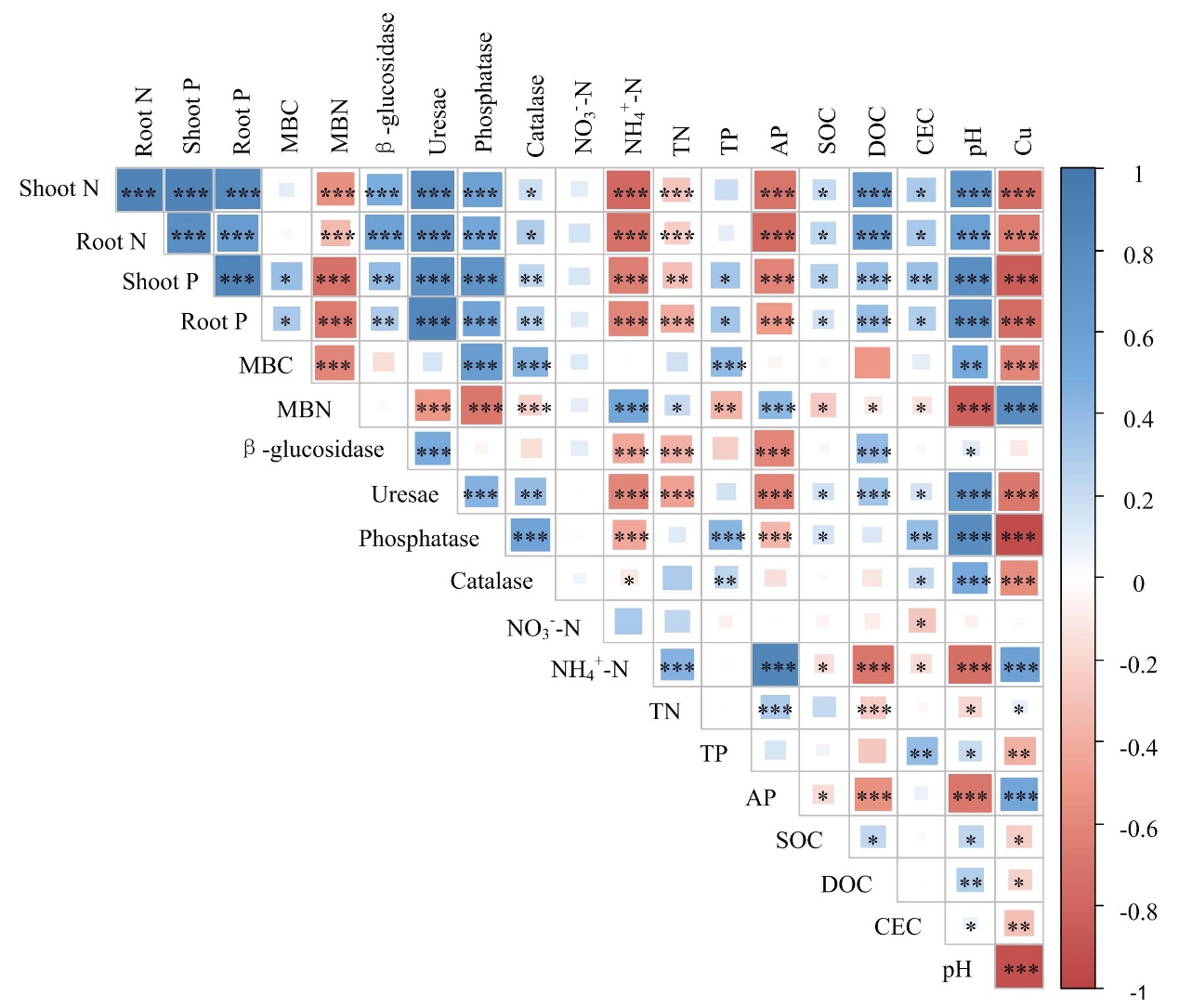


**Fig. S1.** A correlation heat map illustrating pairwise relationships between plant nutrient elements and soil properties based on Pearson correlation analysis. Shoot N: shoot nitrogen concentration; Shoot P: shoot phosphorus concentration; Root N: root nitrogen concentration; Root P: root phosphorus concentration; DOC: dissolved organic carbon; MBC: microbial biomass carbon; MBN: microbial biomass nitrogen; TN: total nitrogen; TP: total phosphorus; AP: available phosphorus; SOC: soil organic carbon; CEC: cation exchange capacity. ***, *P* < 0.001; **, *P* < 0.01; *, *P* < 0.05.
